# Kaposi’s Sarcoma-Associated Herpesvirus K3 and K5 Proteins Down Regulate Both DC-SIGN and DC-SIGNR

**DOI:** 10.1371/journal.pone.0058056

**Published:** 2013-02-27

**Authors:** Sabine M. Lang, Meisha O. F. Bynoe, Roshan Karki, Michael A. Tartell, Robert E. Means

**Affiliations:** Department of Pathology, Yale University School of Medicine, New Haven, Connecticut, United States of America; Hannover Medical School, Germany

## Abstract

Kaposi’s sarcoma-associated herpesvirus (KSHV) is the etiological agent of multicentric Castleman’s disease, primary effusion lymphoma and Kaposi’s sarcoma. In this study, we show that like the C-type lectin DC-SIGN, the closely related DC-SIGNR can also enhance KSHV infection. Following infection, they are both targeted for down modulation and our data indicate that the KSHV MARCH-family ubiquitin ligase K5 is mediating this regulation and subsequent targeting for degradation of DC-SIGN and DC-SIGNR in the context of the virus. The closely related viral K3 protein, is also able to target these lectins in exogenous expressions studies, but only weakly during viral infection. In addition to requiring a functional RING-CH domain, several protein trafficking motifs in the C-terminal region of both K3 and K5 are important in regulation of DC-SIGN and DC-SIGNR. Further exploration of this modulation revealed that DC-SIGN is endocytosed from the cell surface in THP-1 monocytes, but degraded from an internal location with minimal endocytosis in HEK-293 cells. Pull-down data indicate that both K3 and K5 preferentially associate with immature forms of the lectins, mediating their ubiquitylation and degradation. Together, these data emphasize the molecular complexities of K3 and K5, while expanding the repertoire of targets of these two viral proteins.

## Introduction

Kaposi’s sarcoma-associated herpesvirus (KSHV) is a member of the γ2- herpesvirus genus. It is the causative agent of Kaposi’s sarcoma, a cancer of the endothelium, as well as being associated with the B cell lymphoproliferative diseases, multicentric Castleman’s disease and primary effusion lymphoma [1], [2], [3]. As with many pathogens, its genome codes for several protein products that enable it to evade the immune response. Two of these proteins are K3 and K5 (or modulator of immune recognition (MIR) 1 and 2 respectively), coded for by ORF K3 and ORF K5 [4].

K3 and K5, which share approximately 40% identity, have been classified as immediate early gene products [4], [5], [6]. Additionally, both genes may be expressed during latency in response to Notch signaling [7]. K3 and K5 each contain a RING-CH type zinc finger domain at their N-termini, and are the prototypical members of the MARCH (membrane-associated RING-CH containing) family of proteins [8], [9]. These viral proteins, like all of the MARCH family members, have been found to act as E3 ubiquitin ligases, with the RING-CH domain being important for this function [10], [11], [12], [13]. They have been shown to mediate the down regulation of several immunomodulatory proteins, including B7.2 (CD86), intercellular adhesion molecule 1 (ICAM-1; CD54), tetherin (BST-2), IFN-γR and several major histocompatibility complex (MHC) class I haplotypes, as well as additional cellular proteins less tightly linked with immune function, such as CD31 [8], [10], [14], [15], [16], [17], [18], [19], [20]. More recently we have demonstrated that the K5 protein is also able to mediate increased survival and growth signaling through interactions with several receptor tyrosine kinases [21]. While the addition of ubiquitin is playing a clear role in the regulation of the host proteins, the molecular mechanisms controlling protein modulation and degradation are quite complex. Our lab has previously shown that K3 and K5 both contain a number of protein interaction and trafficking motifs that are differentially critical depending on target [11], [22]. Further, some cellular proteins are targeted by the MARCH proteins for endocytosis, some are blocked for exocytosis, and some targets are regulated by multiple mechanisms [11], [21].

DC-SIGN (dendritic cell-specific ICAM-3 non-grabbing integrin, CD209) is expressed on monocytes, macrophages, dendritic cells (DCs) and activated B cells [23], [24], [25], [26], [27], [28]. It has been shown to be important in the activation of the immune response, playing a crucial role in the formation of the immunological synapse, in lymph node homing of DCs, and has been found to be capable of mediating the engulfment of glycoconjugates for later presentation by MHC molecules [23], [29], [30]. DC-SIGN has also been implicated in the polarization of the immune response. Signaling through DC-SIGN while concurrently stimulating various TLRs can block the activation of the TLR-induced type I interferon response [31], [32]. Indeed, DC-SIGN signaling is exploited by pathogens to create an environment conducive to the establishment of productive infection [31], [33], [34], [35]. Finally, it has been shown to act as a receptor for binding and/or entry for several pathogens, including HIV-1, Mycobacterium tuberculosis, dengue virus, ebola virus and more recently, KSHV [12], [27], [28], [30], [36], [37], [38], [39].

In addition to DC-SIGN being used as a receptor, a number of pathogens including West Nile virus and ebola virus use the closely related DC-SIGNR (CD209R) protein as a receptor for infection [40], [41]. DC-SIGNR shares a 73% sequence identity on the nucleotide level with DC-SIGN, but unlike DC-SIGN is found on lymph node and liver sinusoidal endothelial cells [42], [43], [44], [45]. Due to its localization, it is thought that DC-SIGNR may have a role in peripheral tolerance, but its exact role in pathogen responses has not been well characterized.

In this paper, we show that like DC-SIGN, cellular expression of DC-SIGNR increases the efficiency of KSHV infection and following infection it is down regulated. The viral proteins K3 and K5 are candidates for this modulation and we demonstrate using over-expression assays that they are able to target these two lectins for degradation through direct interaction and ubiquitylation. In addition to identifying two new cellular targets for these MARCH family ubiquitin ligases, these data suggest that while aiding in viral immune evasion, K3 and K5 are potentially also enhancing viral release through down regulation of a viral co-receptor.

## Materials and Methods

### Cell culture

HEK 293 K3 and K5 stable cell lines were established by retroviral transduction using the pLXSN vector containing wild-type or mutant K3 or K5 alleles. Stable DC-SIGN or DC-SIGNR expressing HEK 293 cells were established by transfecting the corresponding pcDNA3 expression constructs (kindly provided by Bob Doms, University of Pennsylvannia), followed by G418 selection and subsequently using MACS columns (Miltenyi, Auburn, CA) to select for expressing cells from the cell pool. All HEK 293 and 293T cell lines were maintained in DMEM supplemented with 10% fetal bovine serum (FBS), glutamine (2 mM) and penicillin-streptomycin (100 U/ml; 100 µg/ml). Stable THP-1 cells expressing K3 of K5 constructs were created by retroviral transduction with VSV G protein pseudotyped viruses carrying the gene of interest in the pLXSN vector, as described previously [11]. THP-1 cells were selected with 1 mg of G418 per ml for 4 weeks following transduction and then characterized for construct expression by radioactive IP. All THP-1-derived lines were maintained in RPMI1640 supplemented with 10% FBS, glutamine (2 mM) and penicillin-streptomycin (100 U/ml; 100 µg/ml). Vero rKSHV.219 cells, the kind gift of J. Vieira (University of Washington), were maintained in complete DMEM containing 5 µg/ml puromycin [46]. Stable iSLK puro cell lines carrying BAC16 widltype or deletion mutants of K3, K5 or both were generously provided by Jae Jung (University of Southern California) and maintained as described [47]. Cells were treated with MG132 (Sigma, St. Louis, MO) at a final concentration of 50 µM for 5 hours at 37°C or with chloroquine at a final concentration of 200 µM for 2 hours at room temperature.

To inhibit cellular endocytosis, cells were washed once with serum-free medium and then treated with 80 µM dynasore in serum-free medium for 30 to 60 minutes. To release the dynasore inhibition, FBS was added to a final concentration of 10% and cells transferred onto ice. For endocytosis time courses, cells were pelleted, resuspended in fresh complete medium and incubated for the various time periods at 37°C. At the end of each incubation, sodium azide was added to a final concentration of 0.05% and cells were transferred onto ice before staining for flow cytometry.

### Flow cytometry

Cells were washed in PBS containing 0.5% FBS and 0.1% sodium azide. They were stained with R-phycoerythrin (PE)-, or fluorescein isothiocyanate (FITC)-conjugated antibodies or unconjugated antibodies, as indicated. Incubation with a primary unconjugated antibody was followed by incubation with a PE- or FITC-conjugated secondary antibody. Following fluorescent antibody incubations, the cells were fixed in 4% parafomaldehyde. Cells that were permeabilized prior to staining were first fixed in 2% paraformaldehyde for 15 minutes at room temperature and then permeabilized with saponin buffer (0.5% saponin, 5% FBS, 0.02% sodium azide in PBS) for 10 minutes. Incubations with primary and secondary antibody were done in saponin buffer at room temperature. Following staining, the cells were fixed again in 4% paraformaldehyde. Flow cytometry was performed on a BioRad FACS Calibur machine, followed by analysis using Cell Quest software (BD Biosciences, San Jose, CA) or FlowJo (Tree Star, Inc., Ashland, OR).

### Antibodies

The following antibodies were used: DC-SIGN (MAB161; R&D Systems, Minneapolis, MN), MHC class I (unconjugated or RPE-conjugated W6/32 clone; Dako, Carpinteria, CA), MHC class I-FITC (Serotec, Raleigh, NC), DC-SIGN/R (H-200), DC-SIGNR (N-17), laminB (M-20), α-actinin (C-20), GST (Z-5), GFP (B-2 or FL; Santa Cruz Biotechnology, Santa Cruz, CA); HRP-conjugated donkey anti-mouse, -rabbit, -goat secondary (Santa Cruz Biotechnology); MHC class I (HC-10; obtained from Hidde Ploegh, MIT); and donkey-anti-rabbit-RPE or donkey anti-mouse-RPE secondary (Jackson ImmunoResearch Laboratories, West Grove, PA).

### Plasmid constructs and Transfection

The pDEST27 EglN1 plasmid encoding a GST-tagged EglN1 protein was the kind gift of Dr. Qin Yan (Yale University). All mutations in K3 and K5 have been described previously [11], [21], [22]. For GST pull-down experiments, the indicated constructs were transferred into the pcDEF-GST-AU1 vector. For GFP tagging, constructs were transferred into the pEGFP-N1 vector (Clontech, Mountain View, CA). Transfection of 293 and 293T cell lines was performed using JetPEI (Polyplus-transfection, New York, NY), Lipofectamine 2000 (Invitrogen, Carlsbad, CA) or Transfectin (Bio-Rad, Hercules, CA) according to the respective manufacturer’s recommendations.

### Virus production and titration

Virus was obtained either by reactivation of rKSHV.219 from latently infected Vero cells as described or reactivation of Bac16 wild-type (WT) and mutants viruses from latently infected iSLK puro cells [46], [47]. Culture supernatant was treated by first centrifuging to remove cells, followed by filtration (0.4 µm). Virions were then pelleted at 21,000 rpm through 5 ml of 5% sucrose cushion in a Beckman SW28 rotor at 4°C. Pelleted virion were resuspended in DMEM supplemented with 0.5% FBS in 1/100 of the original volume and used for infection. Virus titers were determined by limiting dilution infection of HEK 293 cells and counting the GFP positive cells 48 hours post infection.

### KSHV infection

The virus-containing filtrate was added to cells plated for infection at a the indicated multiplicity of infection (MOI). The culture plates were centrifuged at 450×g for 20 min and cells were incubated at 37°C for 2 hours, at which point the virus-containing media was replaced with fresh complete DMEM. Cells were harvested 2 to 3 days post-infection.

### Immunoprecipitation, GST pull-down and immunoblot assays

293T or 293 cells were lysed in NP-40 lysis buffer (0.5% NP-40, 150 mM NaCl, 50 mM HEPES pH 7.5) or in RIPA lysis buffer (1% NP-40, 0.5% sodium deoxycholate, 0.1% sodium dodecyl sulfate (SDS) in PBS) containing protease inhibitors (Complete; Roche, Indianapolis, IN) and N-Ethylmaleimide (NEM, 10 mM). To remove insoluble components, the lysates were centrifuged at 16,000×g for 15 minutes at 4°C. Two µg of antibody were used for immunoprecipitation. Immune complexes were adsorbed to protein-A agarose (Santa Cruz Biotechnology) for 3 hours at 4°C while rotating, followed by three washes in lysis buffer. Alternatively, the lysates were incubated with 15 µl of glutathione-sepharose beads (Amersham Pharmacia Biotech AB, Sweden), followed by three washes in lysis buffer. EndoH and PNGaseF digestions were performed according to the manufacturer’s recommendations (New England BioLabs, Ipswich, MA). For immunoprecipitations that were performed on GST pull-down samples, proteins were denatured in 100 µl RIPA buffer containing 1% SDS for 5 min at 95°C. Subsequently, 900 µl of SDS-free RIPA buffer was added followed by a short spin to remove glutathione beads. DC-SIGN was precipitated from the cleared supernatant as described above, with the exception that the DC-SIGN-depleted supernatant was saved for trichloracetic acid (TCA) precipitation (following standard lab protocols) to confirm that GST pull-downs were successful. TCA precipitates were dissolved in 2× SDS sample buffer and processed in parallel with the DC-SIGN immunoprecipitates.

For whole cell lysates (wcl), 293 and 293T cells were lysed in RIPA buffer and protein concentration normalized in SDS sample buffer using the BCA Protein Assay kit (Thermo Scientific, Rockford, IL). Proteins were separated by SDS-polyacrylamide gel electrophoresis (SDS-PAGE) and transferred onto Immobilon-P membrane using a semidry transfer unit (BioRad, Hercules, CA). Membranes were blocked for 1 hour in PBS containing 0.05% Tween-20 and 5% nonfat dry milk (PBS-TM) and then incubated with antibodies diluted in PBS-TM according to the manufacturer’s recommendations. Following incubation in primary antibody for 1.5 hours at room temperature or overnight at 4°C, the blots were washed and incubated for 30 minutes at room temperature in PBS-TM containing the appropriate horseradish peroxidase conjugated secondary antibody. Proteins were detected by enhanced chemiluminescence solution (Millipore, Billerica, MA) using the LAS3000 camera (FujiFilm, Stamford, CT).

## Results

### DC-SIGN and DC-SIGNR can act as co-receptors for KSHV

**Figure 1:**
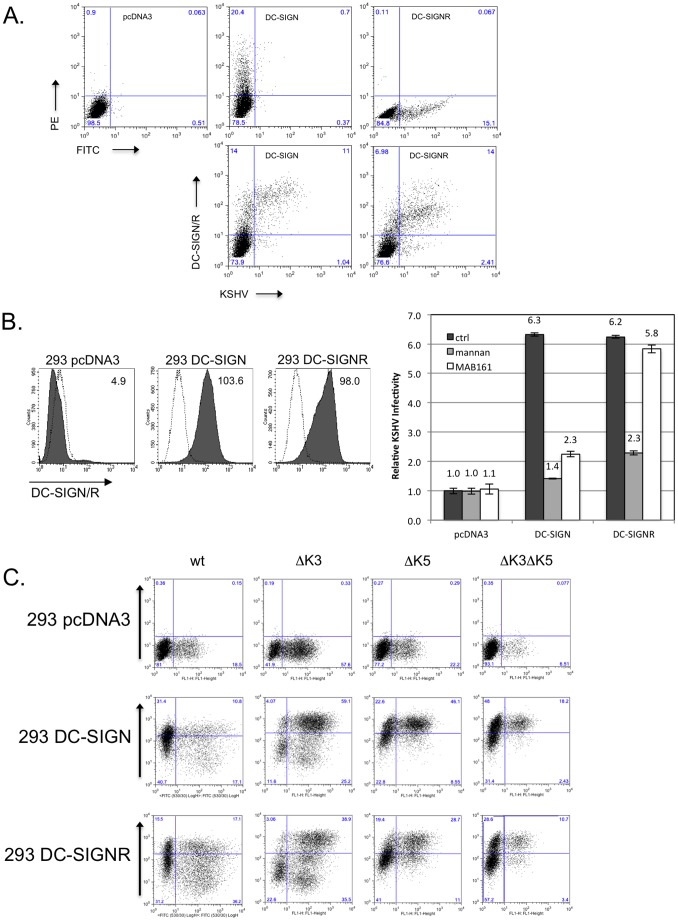
Infectivity of KSHV is enhanced in the presence of DC-SIGN and DC-SIGNR. **A**) 293T cells were transfected with empty pcDNA3 vector or expression constructs for DC-SIGN or DC-SIGNR. After 24 hours, cells were infected with 20 µl Bac16ΔK3ΔK5 or left uninfected as controls. Cells were harvested after additional 24 hours and surface stained with a DC-SIGN/R antibody (H-200) and analyzed by flow cytometry. Top three panels show transfected cells stained for DC-SIGN/R followed by PE- (DC-SIGN), FITC- (DC-SIGNR) or both (vector) conjugated secondary antibodies. Bottom panels shows KSHV infection of 293T cells transiently expressing DC-SIGN or DC-SIGNR. **B, left panels**) 293 cell lines stably expressing a vector construct, DC-SIGN or DC-SIGNR were fluorescently stained for surface expression of DC-SIGN or DC-SIGNR. The mean channel fluorescence is indicated in the upper right hand corner. Open histograms – secondary antibody alone; shaded histograms – DC-SIGN or DC-SIGNR staining. **B, right panel**) 293 pcDNA3, DC-SIGN or DC-SIGNR stable cell lines were pre-incubated with a control antibody (anti-ICAM1, 7 µg/ml), with mannan (100 µg/ml) or a monoclonal antibody specific for DC-SIGN (MAB161; 7 µg/ml) for 30 minutes on ice. These cells were then infected with wild type KSHV (Bac16 or rKSHV.219) at an MOI of 0.01. After 72 hours cells were harvested and evaluated for infection by flow cytometry measuring GFP expression. Infection rates were normalized to 293 pcDNA3 cells treated with the control antibody. The fold increase in relative infectivity is indicated. Data are representative of four independent experiments with two performed in triplicate. **C**) 293 pcDNA3, DC-SIGN or DC-SIGNR stable lines were infected with 50 µl of concentrated Bac16 wildtype (wt), or mutants with deletion of K3 only (ΔK3), K5 only (ΔK5), or deletion of both K3 and K5 (ΔK3ΔK5) as indicated. At 72 hours post-infection, the cells were stained for surface expression of DC-SIGN, DC-SIGNR or MHC class I. GFP fluorescence was used as a marker for infection. MHC I staining is shown for infected 293 DC-SIGNR cells. Inset numbers indicate percentage of cells in each quadrant.

It has been documented that DC-SIGN can act a co-receptor for KSHV, not sufficient for entry by itself, but capable of enhancing viral infectivity [26], [27], [28]. However, the ability of highly homologous DC-SIGNR to act as a receptor for binding/entry of KSHV has not been explored. To begin addressing this deficiency, we transiently transfected 293T cells with empty vector or expression constructs for DC-SIGN or DC-SIGNR. Approximately 24 hours post-transfection (hpt), cells were infected with a low multiplicity of infection (MOI) (0.01) of recombinant KSHV derived from the Bac16 construct, lacking both K3 and K5 genes and also expressing green fluorescent protein (GFP) from a constitutive promoter, or cells were left uninfected as controls. A low MOI was utilized in order to better observe any changes in infection rate that would otherwise be masked by utilizing an excess amount of virus. At 24 hours post-infection (hpi), the cells were collected and stained with an antibody recognizing both DC-SIGN and DC-SIGNR and analyzed by flow cytometry. As shown in Figure 1A, top panels, we were able to achieve nearly identical transfection and cell surface expression of DC-SIGN or DC-SIGNR in the uninfected cells. Strikingly, as shown in the bottom panels, the majority of infection as indicated by GFP fluorescence occurred in the DC-SIGN- or DC-SIGNR-expressing population of cells, with approximately 6–10 fold more infected cells in the upper right quadrant, as opposed to the lower right. These results indicated that DC-SIGN or DC-SIGNR was playing an active role in the increase in viral infection, since cells in the same population that were not expressing either of the lectins, but still exposed to transfection reagent were less efficiently infected.

Transient transfection of the C-type lectins resulted in a large variation of infection enhancement. To address this problem, 293 cells were engineered to stably express DC-SIGN or DC-SIGNR by transfection and antibiotic selection, followed by sorting for highly-expressing populations. Analysis of the these cell lines by flow cytometry showed increased surface levels of either C-type lectin relative to 293 cells stably harboring an empty vector, constructed at the same time (Fig. 1B, left panels). To test each of these cell lines for changes in infectability and the involvement of DC-SIGN or DC-SIGNR in infection enhancement, cells were incubated prior to infection with either mannan, which binds to both DC-SIGN and DC-SIGNR, a monoclonal antibody specific for DC-SIGN only (MAB161) or antibody against ICAM-1 as control, which is not known to have a role in KSHV infection, and should not bind either receptor. These cells were then infected with KSHV, at a MOI of 0.01., again expressing the GFP gene under the control of a constitutive promoter, allowing determination of infection levels by flow cytometry [45]. At 48 hpi, KSHV infection levels were up to 6-fold higher in 293 cells expressing either C-type lectin treated with ICAM-1 antibody, as compared with parental 293 cells receiving the same treatment (Fig. 1B, right panel, black bars). Levels of infection enhancement varied slightly, from ∼5.5 to 9.5 fold, between viral stocks produced at different times, but unlike with transient lectin expression, was consistent within the same stock (Data not shown). Pre-incubation with mannan decreased infection levels in both 293 DC-SIGN and 293 DC-SIGNR stable lines to the levels seen in parental 293 cells (Fig. 1B, right panel, grey bars). However, pre-incubation with the DC-SIGN-specific monoclonal antibody decreased viral infection only in the 293 DC-SIGN stable cells (Fig. 1B, grey bars).

Further examination of the cytometric data revealed that in approximately 25% of cells that expressed higher levels of GFP, and therefore harbored more viral genomes, surface levels of DC-SIGN and DC-SIGNR were diminished compared to uninfected cells or cells with low GFP expression, with a mean channel fluorescence about 50% that of cells expressing low GFP (Data not shown). These data are in agreement with published data indicating DC-SIGN down modulation following KSHV infection [37]. Given the role of the KSHV K3 and K5 proteins in regulating a variety of cell surface molecules, we decided to explore whether they might be playing a role in this regulation.

The same stable cell lines were once again infected, this time with a high MOI of wild type (wt) virus, or previously characterized viruses deleted for the K3 gene (ΔK3), K5 gene (ΔK5) or both (ΔK3ΔK5)[47]. Rather than examining cells at 24 hpi, we harvested and examined cells for GFP and DC-SIGN, DC-SIGNR, or MHC I-expression as a control, at 72 hpi. Once again, we observed increased infection in the presence of either lectin for wt virus (Fig. 1C). This infection enhancement was also observed for each of the other viruses. Each of the viruses encoding a functional K5 gene (wt and ΔK3) showed significant down regulation of DC-SIGN, DC-SIGNR and MHC I from the cell surface. In contrast, the ΔK5 and ΔK3ΔK5 viruses only mediated weak down regulation of any of the examined molecules. The primary publication utilizing these viruses also observed that although K3 protein could be detected in western blot (WB), there was a lack of K3 activity against MHC I, during primary infection, latency, and reactivation. It should be pointed out that interestingly, while MHC I was downregulated in a plurality of infected cells, DC-SIGN and DC-SIGNR regulation only occurred in a subpopulation of cells. Overall, these results indicate that not only is KSHV able to specifically utilize DC-SIGN and DC-SIGNR to increase infectivity, but that it also down regulates both proteins from the cell surface following infection, with K5 playing a dominant role in this context.

### K3 and K5 modulate surface levels of DC-SIGN and DC-SIGNR

**Figure 2:**
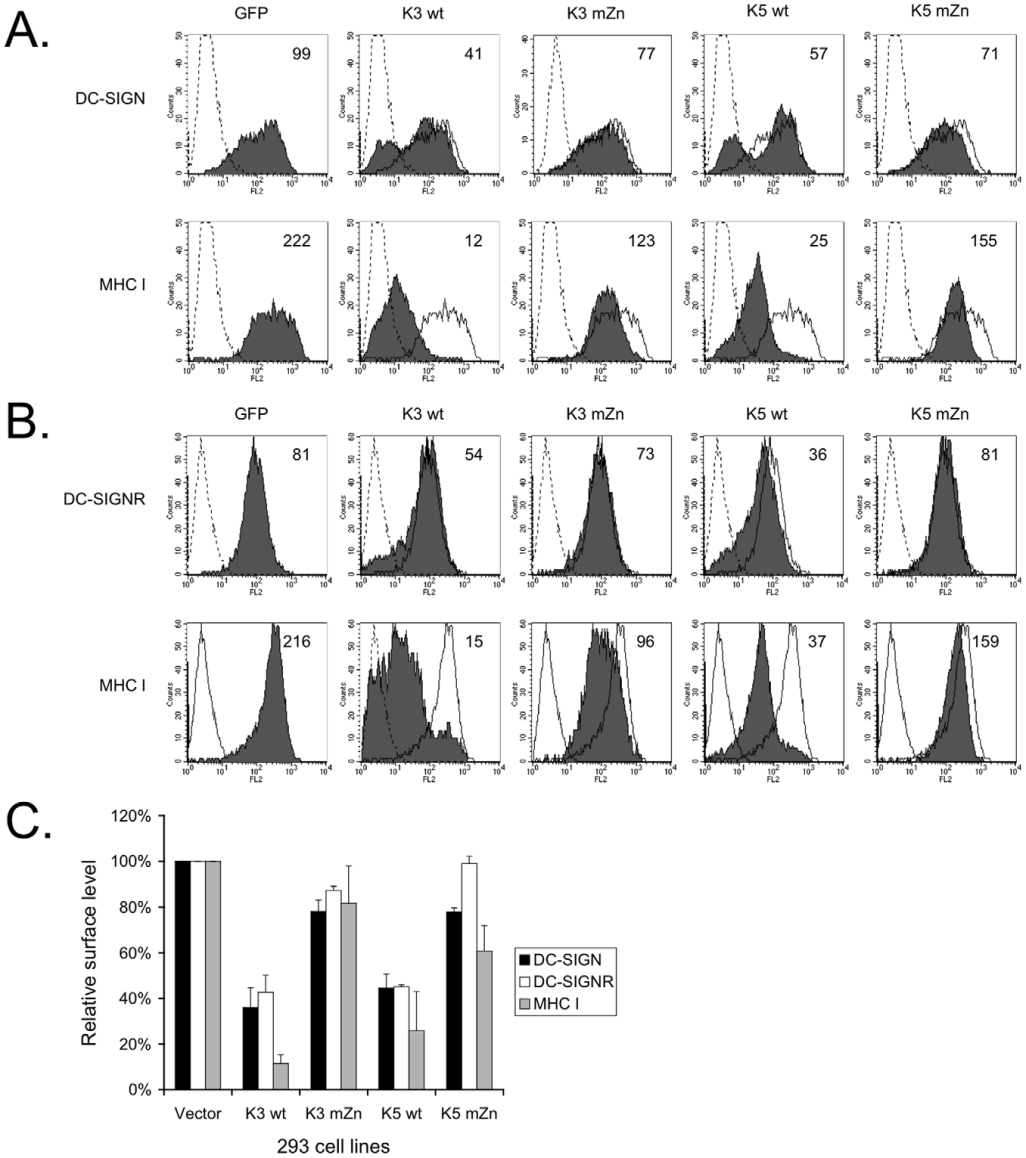
Downregulation of DC-SIGN and DC-SIGNR by viral proteins K3 and K5 is dependent on a functional RING-CH domain. 293 cells stably expressing DC-SIGN or DC-SIGNR were transfected with 4 µg of GFP-tagged expression constructs for wild-type K3 (K3 wt), wild-type K5 (K5 wt), RING-CH mutants (mZn) of each or empty vector. 36–48 hours post-transfection the cells were collected, stained for surface levels of DC-SIGN (**A**), DC-SIGNR (**B**) or endogenous MHC class I (**A and B**), as indicated and subjected to flow cytometry. Live cells were gated for GFP expression and the mean channel fluorescence (MCF) for each was calculated (inset numbers.) The data is representative of at least three experiments. **C**) Flow cytometry data presented in Panels A and B were quantified. The MCF value for each was normalized to vector cells. Error bars indicate standard deviations. The data presented are an average of three independent experiments.

Although we did not see modulation of surface levels of either lectin in the absence of K5, we reasoned that K3 might still be targeting these proteins in other contexts, as is seen with MHC I. To explore this possibility and further examine the ability of K5 to modulate these molecules the 293 DC-SIGN and DC-SIGNR cell lines were transiently transfected with GFP expression constructs fused to either wild-type (wt) K3, wt K5, or variants of either viral gene in which the RING-CH domain had been mutated (mZn) or empty GFP expression vector [11], [22]. At 36–48 hours post-transfection, the cells were harvested and stained for DC-SIGN, DC-SIGNR or MHC I surface expression. Analysis of GFP-positive cells by flow cytometry revealed that, unlike expression in the context of viral infection, both K3 and K5 caused a reduction in surface expression of both DC-SIGN and DC-SIGNR by approximately 60% (Fig. 2). Mutation of the RING-CH domain abolished this effect, with surface levels of DC-SIGN and DC-SIGNR in the presence of K3 mZn or K5 mZn reaching 75–100% of the levels observed in 293 cells expressing vector only. The surface levels of MHC class I in these cells followed a trend analogous to those of DC-SIGN and DC-SIGNR, but generally MHC I was more strongly regulated by both viral proteins. In the presence of wild-type K3 or K5, surface levels of MHC I were reduced about 80–90%. However, in the presence of the RING-CH mutated viral protein, MHC class I levels were only 20–30% lower than in 293 vector cells (Fig. 2). Similar trends indicating the need for a functional RING-CH domain have been previously observed for the other targets of K3 and K5 [10]. We also consistently observed that as with viral infection (Fig. 1C), while the overall level of DC-SIGN or DC-SIGNR in the population was decreased, a sub-population of cells (∼40%) demonstrated far greater down regulation as compared to the overall population, something that was not observed to this extent for MHC I.

### The tyrosine-based endocytosis motif is required in addition to the RING-CH domain to down modulate DC-SIGN and DC-SIGNR in 293 cells

K3 and K5 both possess a number of protein:protein interaction and trafficking motifs within their C-terminal domains, downstream of the second transmembrane domain. In order to deduce whether any of these motifs were important for DC-SIGN or DC-SIGNR modulation, 293 cells stably expressing either C-type lectin were once again transiently transfected with expression constructs coding for GFP-tagged wild-type and mutant K3 and K5 constructs. The mutations included: the previously used mZn, a mutation of initial tyrosine of the tyrosine-based motif to alanine (Y/A) or phenylalanine (Y/F), mutation of the polyproline region (P/A), and mutation of two acidic tracts in the C-terminal domain (DE12) (all described previously [11], [22]). Flow cytometry was used to analyze surface levels of DC-SIGN or DC-SIGNR approximately 36–48 hours post-transfection.

**Figure 3:**
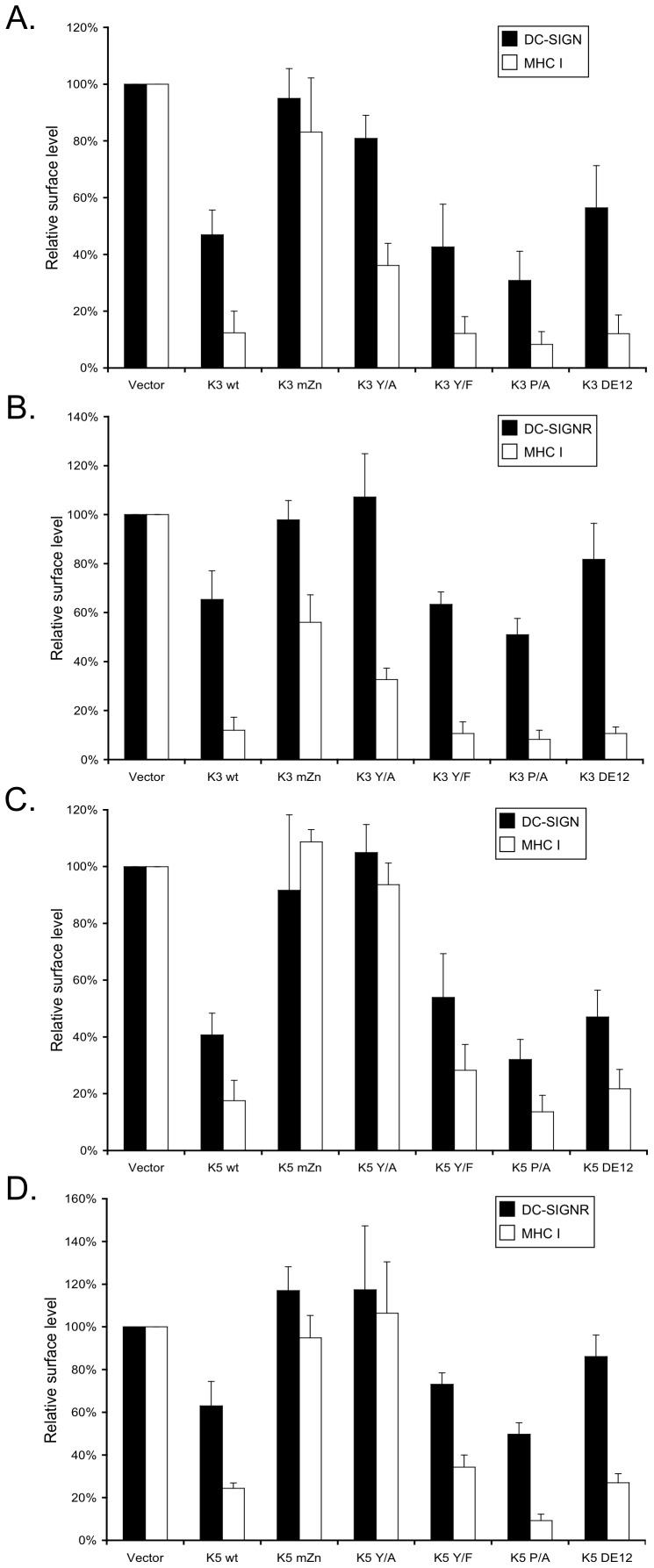
Several motifs of K3 and K5 are required for DC-SIGN modulation. 293 stable cell lines expressing DC-SIGN (panels **A** and **C**) or DC-SIGNR (panels **B** and **D**) were transiently transfected with 4 µg of GFP expression construct for wild-type or the indicated mutants of K3 (panels **A** and **B**), K5 (panels **C** and **D**) or GFP control vector. At 36-48 hours post-transfection, the cells were harvested and surface stained for DC-SIGN, DC-SIGNR, and MHC class I. Using flow cytometry, live cells were gated for GFP expression and the geometic mean channel florescence was normalized to GFP vector expressing cells. The data are an average of three independent experiments and error bars indicate standard deviations.

As shown previously (Fig. 2), mutation of the RING-CH domain in the context of either K3 or K5 abrogated down modulation of both DC-SIGN and DC-SIGNR (Fig. 3). Likewise, the Y/A mutation decreased the ability of both K3 and K5 to modulate surface levels of either C-type lectin in these cells, although mutation of this residue to phenylalanine (Y/F) appeared to have minimal effect on activity. This is in keeping with previous data demonstrating that this motif is acting as an endocytosis motif, not an SH2-binding domain, for targeting of cellular proteins [11]. Equally, K3 and K5 carrying mutations of the polyproline tract (P/A) were still capable of causing decreased surface levels of DC-SIGN and DC-SIGNR. However, mutation of the acidic tracts had differential effects for DC-SIGN and DC-SIGNR. Either MARCH protein containing the DE12 mutation was able to regulate DC-SIGN, but both were significantly reduced in their ability to target DC-SIGNR (Fig. 3A and C versus B and D).

The trend observed for DC-SIGN regulation was generally observed for MHC class I down modulation, with the exception of the regulation of MHC I by K3 Y/A. As noted, both K3 Y/A and K5 Y/A have lost the ability to regulate the C-type lectins, and K5 Y/A has additionally lost the ability to mediate increased endocytosis of MHC I, as reported earlier [11]. K3 Y/A, however, is still able to down regulate cell surface expression of MHC I in 293 cells, potentially due to an ability to sequester MHC I in an intracellular compartment, since it is still able to tightly bind MHC I in pull-down assays (data not shown). These data points to potential differences in both K3 and K5 action, as well as differences in the regulation of these three targets by K3.

### K5 and K3 expression causes enhanced endocytosis of DC-SIGN, but not of DC-SIGNR

**Figure 4:**
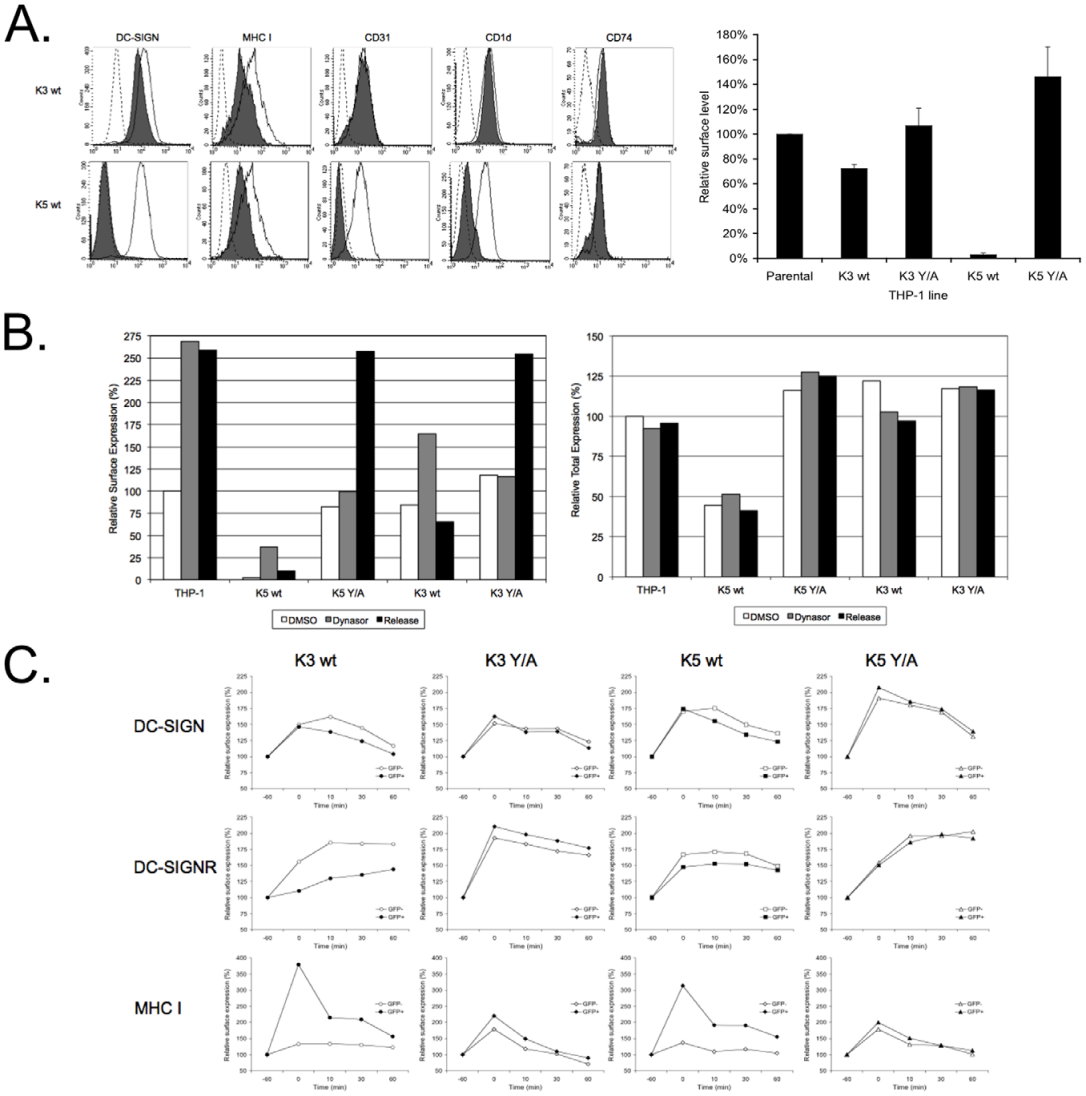
Wild-Type K5 and K3 cause enhanced endocytosis of DC-SIGN, but not DC-SIGNR. **A**) Empty vector transduced THP-1 cells, as well as K3 wt, K3 Y/A, K5 wt and K5 Y/A expressing stable lines, were stained for the indicated surface markers. Histograms on the left show staining of these markers (filled lines) overlayed with isotype control (dashed lines) or with vector transduced THP-1 cells (open lines). As shown in the right bar graph, surface levels of DC-SIGN were determined by flow cytometry and mean channel fluorescence was normalized to vector transduced THP-1 cells. Data shown are an average of three independent experiments with error bars showing standard deviation. **B**) Indicated THP-1 cells lines were treated for 60 minutes with 80 µM dynasore or mock treated with DMSO at 37°C. Upon dynasore removal, cells were chased for 30 min at 37°C in complete medium and endocytosis was stopped by adding sodium azide to all samples. Half of the samples were stained for DC-SIGN surface levels on ice (left panel), while the other half was stained for total DC-SIGN levels after paraformaldehyd fixation and saponin permeabilisation (right panel). Mean channel fluorescence for DMSO treated vector transduced cells was set to 100%. The data shown are representative of three independent experiments. **C**) 293 cells stable expressing DC-SIGN or DC-SIGNR were transiently transfected with 4 µg GFP-tagged K3 wt, K5 wt or the Y/A mutant of either protein. After 36 to 48 hours, cells were subjected to dynasore treatment and release similar to that described in B except that three release time points were assayed. Mean channel fluorescence for surface DC-SIGN, DC-SIGNR, and MHC I were determined by flow cytometry for GFP positive (filled symbols) as well as GFP negative (open symbols) populations. For normalization, DMSO treated cells were set to 100 percent (indicated by the −60 min time point) and relative fluorescence following dynasore treatment (indicated by the 0 min time point) and release (indicated by the 10 min, 30 min and 60 min time points) was calculated. Data presented are representative of three independent experiments.

Published data show that both K3 and K5 are capable of causing increased endocytosis of target proteins, but K5 also targets a sub-set of proteins through altered trafficking from the endoplasmic reticulum or Golgi [11], [48], [49]. Given our finding that K3 or K5 mutated in the tyrosine-based endocytosis motif lost the ability to regulate DC-SIGN and DC-SIGNR, we sought to determine if these lectins were also endocytosed. For these experiments, we first used THP-1 cells that express detectable levels of endogenous DC-SIGN, rather than the over expression cell lines used in earlier experiments. Cell lines stably expressing empty vector, K3 wt or K5 wt were established by retroviral transduction and following selection, were tested for surface expression of a small panel of MARCH protein targets by flow cytometry. As a control, we also examined surface levels of CD74, a protein expressed in THP-1 cells and not known to be regulated by either K3 or K5. Expression of either K3 wt or K5 wt resulted in decreased surface levels of DC-SIGN, although K3 was not as active as K5 in this cell line (Fig. 4A). Examination of RNA derived from these lines by qRT-PCR revealed very low levels of both K3 and K5 mRNA that was ∼2.5 fold greater than the amounts seen in BCBL-1 cells without stimulation, but ∼500 fold less than is seen following reactivation with sodium butyrate and TPA. Regulation of MHC I was found to be equivalent between K3- and K5-expressing THP-1 cells, indicating that the differences in DC-SIGN regulation were likely not due to differences in MARCH protein expression. Both CD31 and CD1d, targets of K5, but not K3, were efficiently regulated in the THP-1 K5 wt cell line. As expected, no modulation of CD74 was observed for either of the wild-type MARCH proteins.

To further extend these observations, we established two additional cell lines expressing the K3 Y/A or K5 Y/A constructs. Mutation of the tyrosine-based motif in either K3 or K5 abolished the ability of the respective protein to down modulate DC-SIGN, in keeping with the modulation observed in 293 DC-SIGN stable cell lines transiently expressing either the K3 Y/A or K5 Y/A mutant (Fig. 4A, compared with Figs. 2 and 3).

In order to examine potential endocytosis in the presence of K3 or K5, we utilized dynasore, a reversible dynamin inhibitor. Cells were treated with either dynasore or solvent (DMSO) for 1 hour. Following treatment, each of the cell lines were stained, on ice, with anti-DC-SIGN antibody, and then chased for 30 minutes at 37°C. Each set of cells was stained with a fluorophore-conjugated secondary antibody and the level of DC-SIGN remaining on the surface was determined by flow cytometry and normalized to the amount found in solvent-treated cells. Treatment with dynasore resulted in a 1.5- to 2.5-fold increase in DC-SIGN surface levels in the parental and K3 wt cell lines (Fig. 4B left panel). In the THP-1 K5 wt cells, an increase of at least 5-fold was observed in multiple experiments, although the levels of DC-SIGN on the surface still remained far below that of the parental cell line. These increases were the result of arrest of DC-SIGN at the cell surface, rather than increases in total DC-SIGN proteins concentrations, as evidenced by flow cytometry of the same cell populations after permeabilization to label both surface and intracellular proteins (Fig. 4B, left panel). The K3 YA- and K5 YA-expressing cells did not show a comparable increase in cell surface DC-SIGN levels at early time points following dynasore treatment, reflecting delayed transport of DC-SIGN to the surface, again likely mediated through their ability to bind, but not drive endocytosis or degradation.

In the presence of K5, accumulated DC-SIGN was endocytosed following dynasore removal, with cell surface levels decreasing ∼60%, but total protein levels remaining static (Fig. 4B, left vs. right panel). K3 expression also caused increased endocytosis of DC-SIGN relative to vector expressing cells, with surface levels decreasing ∼60% during the 30 minute chase. Interestingly, DC-SIGN accumulated at the surface in both the K3 Y/A and K5 Y/A cells only at later time points with Dynasore washout having no impact, as was seen with the vector expressing cells. Additional experiments revealed that this accumulation at the surface occurred at a later time point with or without Dynasore washout (Data not shown). Overall, this indicates both that the endogenous rate of DC-SIGN endocytosis in THP-1 cells is low, and that the Y/A mutants of both viral MARCH proteins have impacts on DC-SIGN egress, but not endocytosis.

In addition to looking at endocytosis in THP-1 cells, we opted to evaluate the activity of K3 and K5 in 293 cells stably expressing DC-SIGN and DC-SIGNR. We transiently transfected these cells with GFP-tagged K3 wt, K5 wt or the Y/A mutants of either viral protein. At 36–48 hours post-transfection, we treated the cells with dynasore and assayed the rate of endocytosis in these cells, as was done with the THP-1 lines. We compared the endocytosis rates of DC-SIGN, DC-SIGNR and as control, MHC class I, in cells that were GFP-positive (transfected) compared with those that did not express GFP (non-transfected) in the same cultures. In this experiments, we observed a minimal, but significant acceleration in endocytosis of DC-SIGN in the presence of wild-type K3 and wild-type K5 (Fig. 4C, top panels). This difference was abrogated by the introduction of the Y/A mutation into either viral protein. No endocytosis was observed, however for DC- SIGNR (Fig 4C, middle panels). In the presence of wild-type K3 wt there was a significant delay to cell surface appearance of DC-SIGNR following dynasore addition. Additionally, the levels of DC-SIGNR surface protein never reached the levels seen in non-transfected cells from the same culture. While this delay was not as pronounced for wild-type K5, cell surface accumulation in the GFP+ population was less rapid than in the GFP- population. Additionally, only minimal decreases in the amounts of cell surface DC-SIGNR were observed over the time course, again indicating a lack of endocytosis. This relative delay in or lack of an increase in DC-SIGNR surface levels was alleviated in the presence of K3 Y/A and K5 Y/A. These observations were not due to a defect in the endocytic machinery in the transfected cells, as MHC class I was rapidly endocytosed the presence of either wild-type K3 or K5 (Fig. 4C, bottom panels). Given the apparent differences in regulation between 293 and THP-1 cells, these data underscore the need to examine the cellular context for each MARCH ligase target and not just the targets themselves.

### K3 and K5 expression affect DC-SIGN and DC-SIGNR stability

**Figure 5:**
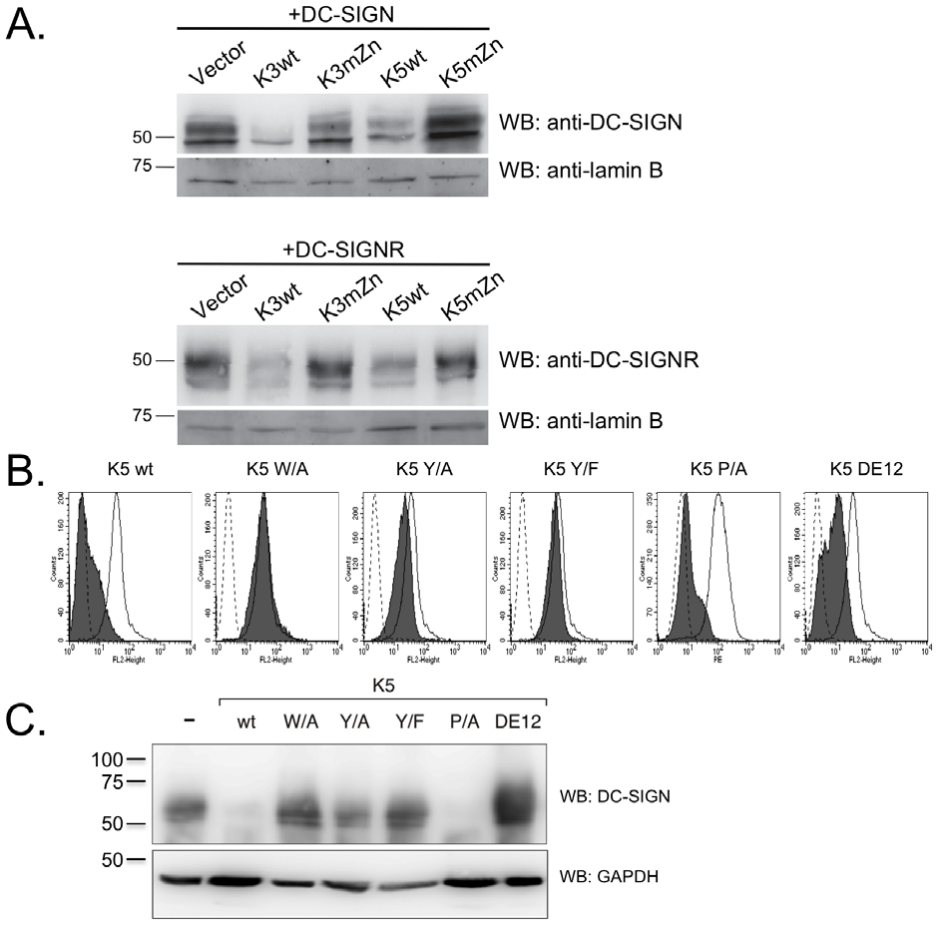
K3 and K5 affect the stability of DC-SIGN and DC-SIGNR in a RING-CH domain-dependent mechanism. **A**) 293 cells stably expressing wild-type K3 or K5, or the RING-CH mutant of either viral protein were transiently transfected with 2 µg DC-SIGN or DC-SIGNR constructs. At ∼48 hpt,cells were lysed in RIPA buffer and 30 µg of normalized lysate were loaded per sample. Protein levels of DC-SIGN or DC-SIGNR were determined by WB, and then blots were reprobed for lamin B as a loading control. Data is representative of at least three independent experiments. **B**) THP-1 cell stably expressing the indicated K5 constructs or empty vector were stained for cell surface levels of DC-SIGN. Solid histogram, empty vector; grey histogram, K5 construct; dotted histogram, isotype control. **C**) The same THP-1 cell lines used in Panel B, were lysed and subjected to western blotting with either a DC-SIGN (H-200) or GAPDH (O411) antibody, as loading control. Data is representative of at least three independent experiments.

It is possible that the reduced surface expression of DC-SIGN and DC- SIGNR in the presence of wild-type K3 and K5 might have been due only to removal from the cell surface, as with CD1d, or due to both removal and targeting for degradation, as for MHC I [14], [19], [50]. To test if K3 and K5 were able to target DC-SIGN or DC-SIGNR for degradation, we transiently transfected 293 cells stably expressing K3 wt, K5 wt, K3 mZn, K5 mZn or carrying empty vector with expression constructs for DC-SIGN or DC-SIGNR. At 48 hours post- transfection, the cell lysates were subject to western blotting (WB), probing for DC-SIGN or DC-SIGNR. As shown in figure 5A, expression of either wild-type K3 or K5 resulted in decreased protein levels of both DC-SIGN and DC-SIGNR. Mutation of the RING-CH domain in either K3 or K5 abrogated this ability.

Given the differences in regulation between 293 and THP-1 cells that we describe above, we also examined levels of endogenous DC-SIGN by flow cytometry and western blot in THP-1 cell lines stably expressing the different K5 variants. In keeping with results from the 293 cell lines, K5 wild-type and K5 P/A-expressing THP-1 cells lines were able to substantially decrease surface levels of DC-SIGN, while the cells expressing the K5 DE12 mutant only demonstrated a partial down-regulation as measured by flow cytometry (Fig. 5B). Cells expressing a K5 variant carrying a mutation of the conserved tryptophan (residue 46) in the RING-CH domain to alanine (K5 W/A), which impairs E3 ubiquitin ligase activity (data not shown), or the K5 Y/A or Y/F mutants showed no significant surface modulation of DC-SIGN and resembled cells expressing empty vector.

To determine total DC-SIGN protein levels, normalized whole cell lysates from each of the stable cell lines were examined by WB. DC-SIGN protein levels in these cells essentially mirrored the flow cytometry results, with decreases seen for wild-type and the K5 P/A constructs, and no change in protein levels for the K5 W/A, Y/A and Y/F-expressing cell lines (Fig. 5C). The K5 DE12-expressing cell lines contained a high level of DC-SIGN protein even though cell surface levels are lower than in cells expressing empty vector. This is in keeping with earlier observations that this domain is involved in MHC class I protein degradation, but not with MHC class I internalization [11], [22].

### DC-SIGN and DC-SIGNR co-precipitate with K3 and K5

**Figure 6:**
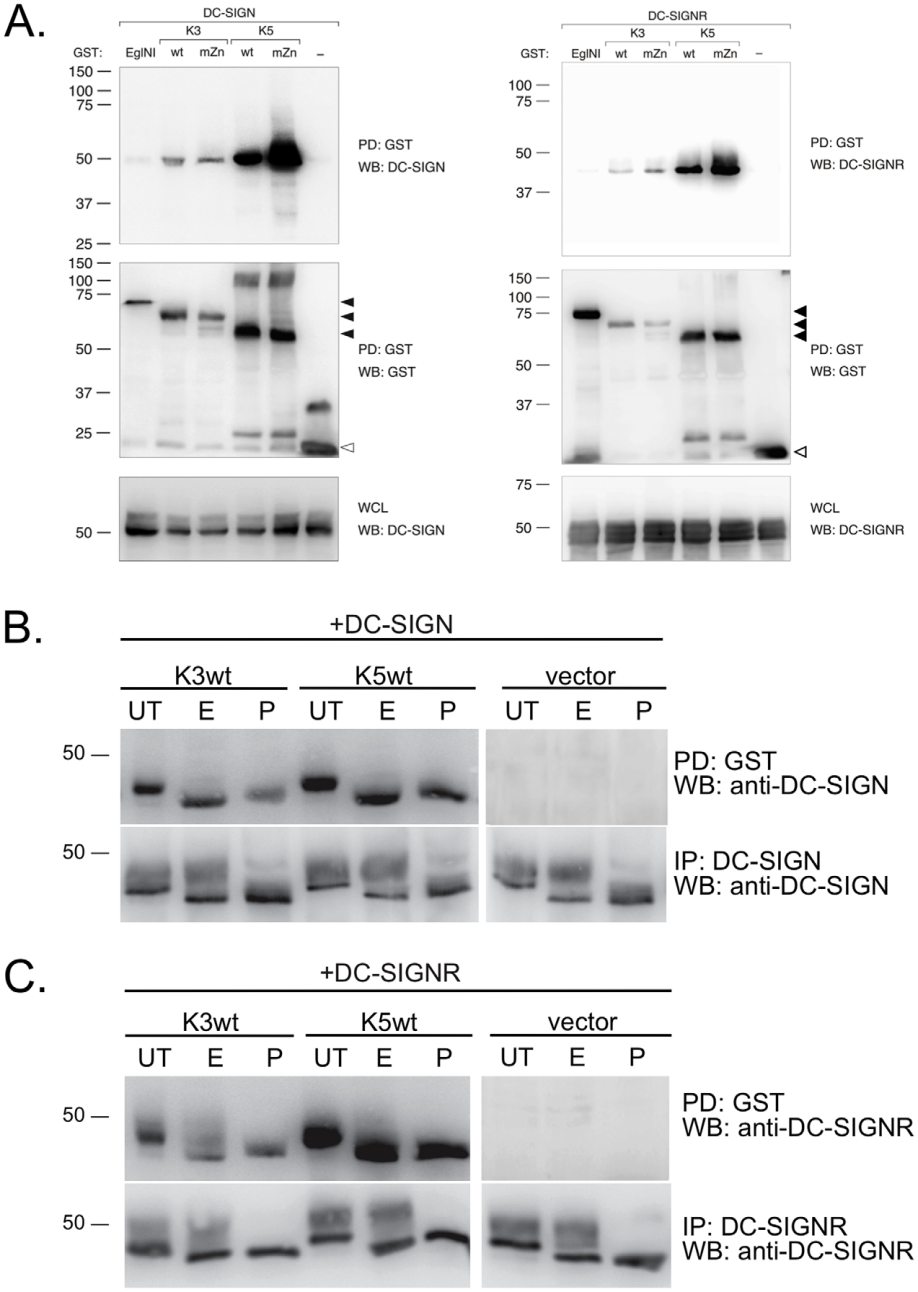
DC-SIGN and DC-SIGNR co-precipitate with K3 and K5. **A**) 293T cells were transiently transfected with 1 µg DC-SIGN (left panels) or DC-SIGNR (right panels) together with 3 µg wild type (wt) or RING-CH mutant (mZn) constructs of GST-tagged K3 or K5, and as controls EglN1, an unrelated protein, and empty GST expression vector (−) as indicated. At 36–48 hours post-transfection, cells were harvested, lysed in NP40 lysis buffer and subjected to pull-down (PD) using glutathione-sepharose beads. Purified proteins were resolved by SDS-PAGE electrophoresis and subjected to WB analysis for DC-SIGN/R co-purification. GST pull-down was evaluated by reprobing the blots with a GST specific antibody. Even expression of DC-SIGN or DC-SIGNR was verified by WB of whole cell lysates (WCL).Closed arrowheads indicate GST-tagged EglNI, K3 or K5; open arrowheads indicate unfused GST. Data is representative of multiple experiments. **B and C**) Co-transfection of 293T cells with (**B**) DC-SIGN or (**C**) DC-SIGNR together with vector or GST-tagged wild-type K3 or K5 was repeated. Samples were split in half and subjected to either GST pull-down or immunoprecipitation for lectin proteins. Aliquots of the purified proteins were left untreated (UT) or digested with either EndoH (E) or PNGaseF (P). Following SDS-PAGE, changes in mobility were detected by WB for DC-SIGN or DC-SIGNR. Data is representative of multiple experiments.

To further examine the mechanisms of DC-SIGN and DC-SIGNR regulation by K3 and K5, we decided to test the ability of either cellular protein to co-precipitate with viral proteins. Given that K3 and K5 cause degradation of DC-SIGN and DC-SIGNR, we decided to look at the RING-CH mutant as well, as it is not able to target DC-SIGN or DC-SIGNR for degradation, allowing us to potentially better visualize the target protein by WB following co-precipitation. We co-transfected 293T cells with DC-SIGN or DC-SIGNR in combination with an empty GST vector (−), an unrelated protein, EglNI tagged with GST or GST-tagged K3 wt, K3 mZn, K5 wt or K5 mZn. Pull-down of GST-tagged proteins followed by WB showed that both DC-SIGN and DC-SIGNR co-precipitated with both K3 and K5 (Fig. 6A, upper panels). A functional RING-CH domain was not necessary for pull-down of either cellular protein with K3 or K5. Re-probe of the blot with an anti-GST antibody confirmed pull-down of the various MARCH proteins, as well as the controls (Fig. 6A, middle panels). Examination of WCL by WB with antibody against DC-SIGN or DC-SIGNR similarly revealed that the lack of interaction with the EglN1 and empty vector was not due to a lack of lectin expression (Fig. 6A, bottom panels).

Western blotting for DC-SIGN and DC-SIGNR in whole cell lysates typically resulted in the visualization of two bands (Fig. 6A, lower panels). In our co-precipitation experiments, however both K3 and K5 appeared to preferentially pull-down the a single band corresponding to the higher mobility form of DC-SIGN and DC-SIGNR. We postulated that the presence of two bands was due to the glycosylation status, and if that was indeed the case, the viral proteins were primarily interacting with the EndoH- sensitive, immature form of DC-SIGN or DC-SIGNR. To validate our hypothesis, we repeated the co-precipitation assay of DC-SIGN and DC-SIGNR with K3 wt or K5 wt, followed by mock treatment or digestion with EndoH or PNGaseF. As controls, we directly immunoprecipitated either DC-SIGN or DC-SIGNR from a parallel aliquot of these cells followed by EndoH and PNGaseF digestion. We found that K3 or K5 preferentially allowed for pull-down of an EndoH-sensitive form of DC-SIGN and DC-SIGNR and PNGaseF treatment did not further alter the mobility (Fig. 6B and C, upper panels). We found that, in cells transfected with either C-type lectin the immunoprecipitated protein was visualized as two bands independent of K3 or K5 co-expression. The higher mobility proteins proved EndoH-sensitive whereas the less rapidly migrating proteins were EndoH- resistent consistent with immature and mature forms of DC-SIGN and DC- SIGNR, respectively (Fig 6B and C, lower panels). From these experiments, we concluded that the initial interactions between the viral proteins and DC-SIGN or DC-SIGNR is likely to occur in a pre-Golgi compartment with immature forms of these two lectins.

### K3 and K5 cause ubiquitylation and proteasomal/lysosomal-dependent degradation of DC-SIGN

**Figure 7:**
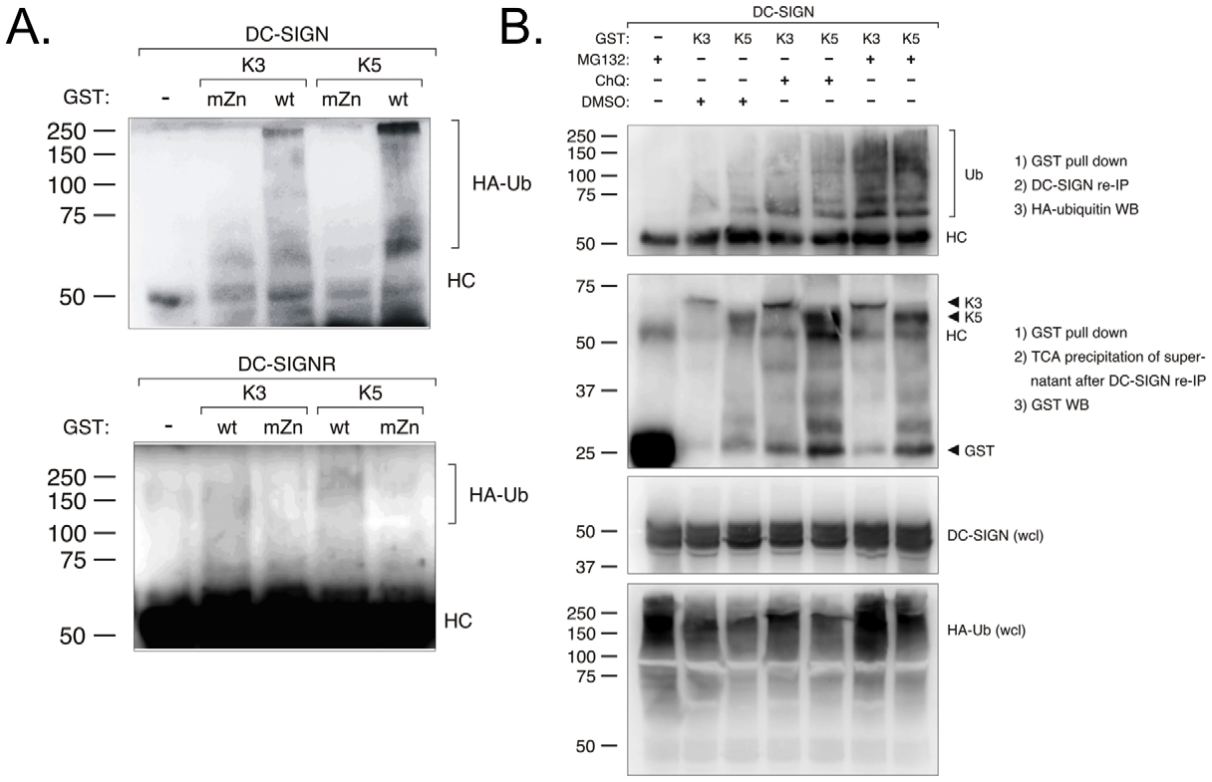
DC-SIGN and DC-SIGNR are ubiquitylated and degraded in a proteasomal- and lysosomal-dependent pathway by K3 and K5. **A**) 293T cells were co-transfected with either 1.75 µg DC-SIGN (top panels) or 1.75 µg DC-SIGNR (bottom panels), 0.5 ug HA-tagged ubiquitin, and 1.75 µg wild-type or RING-CH mutant constructs of GST-tagged K3 or K5, as indicated. At 36–48 hours post-transfection, cells were collected and lysed. Pull-down (PD) was done using glutathione-sepharose beads, followed by SDS-PAGE and immunoblotting analysis. The precipitated proteins were probed with anti-HA antibodies. **B**) 293T cells were transiently transfected as in panel A. At 36–48 hours post-transfection cells were treated with chloroquine (ChQ ), MG132, or DMSO (solvent), as indicated. Cell lysates were then subjected to pull-down with glutathione beads followed by immunoprecipitation of DC-SIGN. Precipitated proteins were then subjected to western blot with an anti-HA antibody. Supernatants from the immunoprecipitation were subjected to TCA precipitation and then western blot with an anti-GST antibody. As additional controls, whole cell lysates were subjected to western blot with antibodies against DC-SIGN or HA-tagged ubiquitin. Data is representative of multiple experiments.

Given that both DC-SIGN and DC-SIGNR were down regulated by K3 and K5, but not mutants containing a non-functional RING-CH domain, we reasoned that the MARCH family ligases were mediating ubiquitylation of both lectins. To address this question, we co-transfected 293T cells with empty vector, GST-tagged wild-type or mZn K3 or K5 constructs together with constructs for DC- SIGN or DC-SIGNR and HA-tagged ubiquitin. After 48 hours we performed a GST pull-down followed by an immunoprecipitation for either DC-SIGN or DC-SIGNR. The precipitated proteins were then probed for the presence of ubiquitin in WB using an anti-HA-antibody (Fig. 7, panel A). We confirmed that DC-SIGN displayed ubiquitylation only in the presence of K3 or K5 with an intact RING-CH domain and not in the presence K3 mZn or K5 mZn. We consistently were able to visualize a much lower level of ubiquitylation of DC-SIGNR, even though relatively equal co-precipitation was achieved (Fig. 7A, and data not shown). However, once again, the presence of the ubiquitylated protein was dependant on the presence of an intact RING-CH domain.

Finally, we examined whether this modification by K3 or K5 led to a degradation of DC-SIGN by a lysosomal or proteasomal mechanism. Again, 293T cells were co-transfected with expression construct for DC-SIGN and HA- ubiquitin together with empty GST vector, GST-tagged K3 or K5. At 48 hours post-transfection cells were treated either with DMSO as control, the lysosomal inhibitor chloroquine, or the proteasomal inhibitor MG132. Cells were then subjected to GST pull-down followed by immunoprecipitation with an anti-DC-SIGN antibody and then western blot with anti-HA antibodies (Fig. 7B, top panel). Again, ubiquitylated DC-SIGN protein was seen in the presence of either K3 or K5. The amount of visualized protein was slightly increased in the presence of chloroquine, while MG132 treatment of the cells very significantly increased ubiquitylation. As a control to insure equal pull-down of the GST-tagged K3 or K5 proteins in the presence of the inhibitors, following DC-SIGN immunoprecipitation the supernatant was subjected to TCA precipitation and western blot with an anti- GST antibody (Fig. 7B, middle panel). Equally, overall levels of DC-SIGN and HA-tagged ubiquitin were examined in whole cell lysates and found to be comparable in all of the samples (Fig. 7B, bottom two panels). Neither treatment was effective at increasing the amount of ubiquitylated DC-SIGNR protein in parallel experiments (data not shown). These results indicate that degradation of DC-SIGN occurs primarily through a ubiquitylation-dependent, proteasomal mechanism, but that a fraction of DC-SIGN might be degraded through a lysosomal pathway. The mechanism of DC-SIGNR degradation remains unclear from these experiments.

## Discussion

The KSHV proteins K3 and K5 have been implicated in down regulation and degradation of a number of molecules important in activation of the immune response, contributing to persistence in the infected host. In this study, we have presented data indicating that expression of DC-SIGNR, like the previously published DC-SIGN, is able to increase infection by KSHV, and that following infection, both DC-SIGN and DC- SIGNR are down modulated from the cell surface [26], [27]. We have shown that the KSHV proteins K3 and K5 can, in the correct context target both DC-SIGN and DC-SIGNR for down modulation and degradation, and that a number of motifs within the viral proteins are important for this phenomenon to occur. Further, we demonstrate that the viral MARCH proteins interact primarily with an immature form of the two C-type lectins in 293 cells, localizing their action to the endoplasmic reticulum or very early Golgi. Going forward, examining and understanding the role of these two viral ligases and KSHV infection in primary cells expressing endogenous DC-SIGN or DC-SIGNR, such as specific endothelial cells or activated B lymphocytes, will be important. These types of experiments might yield information concerning the populations of cells in these experiments that don’t demonstrate down regulation following either infection or exogenous K3 or K5 expression.

KSHV has been shown to infect monocytes, macrophages and DCs, all of which express DC-SIGN. As DC-SIGN is important in immunological synapse formation and antigen uptake, it is likely that by reducing DC-SIGN levels in these antigen presenting cells, K3 and K5 will have inhibitory effects on activation of T cells. Additionally, further modulation of the immune response may be mediated through modulation of DC-SIGN signaling pathway. Mycobacterium and West Nile virus, for example, have been shown to trigger DC-SIGN signaling to alter or block DC maturation, cytokine production and TLR signaling [32], [51], [52], [53]. We are therefore currently examining the signaling cascade that results following DC-SIGN ligation by KSHV, and how expression of K3 or K5 might affect this signal and thus alter monocyte differentiation, DC maturation or cytokine responses during KSHV infection.

An equally exciting possibility is that the virus targets DC-SIGN and DC- SIGNR for regulation of viral entry and egress. Given a role for both lectins in entry, down modulation would reduce superinfection. A similar phenomenon has been observed for HIV, which causes down modulation of the receptor CD4 following infection [54]. The possibility also exists that DC-SIGN and DC-SIGNR down modulation enhances viral egress, as has been demonstrated in the case of tetherin. Here, K5 expression was shown to cause the ubiquitination and subsequent degradation of this cellular protein, permitting virion release [18], [55]. We are currently examining viral egress using K3 and K5 deletion viruses to see if DC-SIGN or DC-SIGNR can also act to restrict virus release through trapping of particles in the absence of the viral MARCH proteins. Given the relatively weak down modulation of these lectins by K3 in the context of viral infection (Fig. 1C), further studies to examine other cell types and changes in K3 expression or modification during viral reactivation and replication are warranted.

Modulation of targets of K3 and K5 can occur via several mechanisms – CD1d is down modulated from the surface but not degraded, unlike MHC class I that is endocytosed from the surface and targeted for degradation, while CD86 is targeted both at the surface and from an internal compartment for degradation [11], [15], [19], [50]. In the case of DC-SIGN, it appears that both K3 and K5 can target the cellular protein for endocytosis and degradation in THP-1 cells (Fig. 4B, 5C, and data not shown). However, the dynasore treatment of 293 cells stably expressing DC-SIGNR in particular points to there being more to the mechanism than simple down modulation from the surface. For both K3 and K5 there was a decreased accumulation of DC-SIGNR on the surface following dynasore treatment, and very little increase in the rate of endocytosis (Fig. 4C). This was especially noticeable for the expression of wild-type K3 in 293 DC-SIGNR cells where cell surface lectin changed only marginally following dynasore treatment or washout. This likely indicates that the wild type viral MARCH proteins are acting to inhibit DC-SIGNR exocytosis in 293 cells and that the Y/A mutants might similarly be inhibiting transport of these lectins to the cell surface in THP-1 cells (Fig. 4B). Exploration of regulation of DC-SIGNR in a more physiological cell type was hampered by a lack of cell lines expressing endogenous levels of this lectin, but this should be explored in the future to establish if this difference in mechanism is completely due to target differences, or if cell type also has an influence. These results, once again underscore that K3 and K5 are not utilizing identical mechanisms to target each cellular protein, but tapping into different pathways that may reflect aspects of the target protein biology.

The hypothesis that DC-SIGN and DC-SIGNR can be targeted from an internal compartment is supported by co-precipitation data presented here. We have been able to show that both DC-SIGN and DC-SIGNR can be pulled down in complex with either K3 or K5 (Fig. 6A). A higher mobility, endoglycosidase- H (EndoH) sensitive form of either C-type lectin appeared to associate preferentially with the viral proteins (Fig. 6B and C). These data suggest that both K3 and K5 target DC-SIGN and DC-SIGNR from the ER or early Golgi, as appears to be the case with modulation of B7.2 by K5 and MHC class I heavy chain, TAP and tapasin by the murine gammaherpesvirus-68 homolog mK3 [11], [56], [57], [58], [59]. Unpublished data, alongside data presented here, indicate however that some synthesized DC-SIGN and DC-SIGNR is able to reach the surface before being targeted for downmodulation by K3 or K5. Interestingly, it has previously been shown that K5 can target the nonclassical MHC I-related molecule HFE both from the cell surface and from the Golgi or a post-Golgi compartment within the cell [49]. K5-associated HFE, in that case, was found to be EndoH insensitive. This points to yet another level of complexity for K5 targeting of cellular proteins for destruction. While the mechanism of K5 targeting from different compartments is unclear, it could be related to the polyubiquitin linkage, additional modifications found on the K5 protein, or non-cannonical ubiquitylation of cysteine residues within the target and we are currently exploring these possibilities [58], [60].

We found that several motifs within K3 and K5 are necessary for modulation of DC-SIGN and DC-SIGNR. In addition to requiring a functional RING-CH domain, both MARCH proteins require a functional tyrosine-based motif. Mutation of the initial tyrosine (residue 152 in K3 and 156 in K5) to alanine or phenylalanine substantially reduced cell surface modulation of both DC-SIGN and DC-SIGNR. Interestingly, K3 with an alanine substitution in this motif is still able to modulate MHC I in 293 cells, while the analogous K5 mutant is not (Fig. 3A and B versus C and D). Thus, even though these two proteins share high structural and domain homology, at a molecular level they might function quite differently. Similarly, while the K5 DE12 construct was fully competent for regulation of DC-SIGN in 293, it was impaired in THP-1 cell (Fig. 3C versus 5B). This indicates that K5 is likely pirating different members of the endocytic machinery depending on cell type. Since KSHV is able to infect a wide variety of cell types, this points out a need to examine and compare the molecular mechanisms of action for these two ubiquitin ligases in a wider variety of cells in the future.

In summary, we have demonstrated that DC-SIGN and DC-SIGNR are both down modulated and targeted for degradation by KSHV following infection. We have explored the mechanisms of action of the two viral proteins capable of mediating this down regulation, K3 and K5. Our data show that, although both K3 and K5 target DC-SIGN and DC-SIGNR, the regulation of each lectin occurs through a slightly different mechanism. K3 and K5 appear to cause down modulation of DC-SIGN from the surface of both THP-1 and 293 cells, with K5 having greater activity, as evidenced by the marked increase in DC-SIGN accumulation in the presence of dynasore and the increased rate of endocytosis. Conversely, the viral ligases do not appear to affect endocytosis rates of DC-SIGNR in 293 cells. However, both proteins likely also deliver immature DC-SIGN and DC-SIGNR to a degratory compartment, blocking exocytosis of these two C-type lectins.

Given the fact that both K3 and K5 need to retain E3 ubiquitin ligase activity for modulation, but not interaction, also has implications for cellular breakdown of immature proteins. In subsequent work, we hope to delve further into the mechanisms of K3 and K5 activity and to determine the immunological consequences of the targeting of DC-SIGN and DC-SIGNR recorded here.
